# Low skeletal muscle density is independently associated with cardiac valve calcification in dialysis patients

**DOI:** 10.3389/fphys.2025.1690904

**Published:** 2025-12-18

**Authors:** Meng-Ting Li, Jing-Yuan Cao, Min Li, Zhen Zhao, Jia-Run Mi, Min Yang, Liu-Ping Zhang, Zi-Fan Wang, Tian-Ke Yu, Ping-Ping Ju, Yu-Jia Jiang, Yao Wang, Xiao-Xu Wang

**Affiliations:** 1 Department of Nephrology, Zhongda Hospital, Southeast University School of Medicine, Nanjing, China; 2 Department of Nephrology, The Affiliated Taizhou People’s Hospital of Nanjing Medical University, Taizhou School of Clinical Medicine, Nanjing Medical University, Taizhou, China; 3 Department of Nephrology, The Third Affiliated Hospital of Soochow University, Soochow University, Changzhou, China; 4 Jiangsu Key Laboratory of Molecular and Functional Imaging, Department of Radiology, Zhongda Hospital, Southeast University School of Medicine, Nanjing, China; 5 School of Health Policy and Management, Chinese Academy of Medical Sciences and Peking Union Medical College, Beijing, China; 6 Department of Nephrology, The Affiliated Hospital of Yangzhou University, Yangzhou University, Yangzhou, China; 7 Department of Nephrology, Qilu Hospital of Shandong University, Shandong University, Jinan, China

**Keywords:** skeletal muscle density, muscle quality, cardiac valve calcification, cardiovascular diseases, dialysis

## Abstract

Assessing muscle characteristics is an emerging field for improving stratification of cardiovascular disease risks. However, the relationship between muscle characteristics and cardiac valve calcification (CVC) remains unclear. This study evaluated how muscle mass and muscle quality relate to the risk of CVC in dialysis patients. This study included dialysis patients from four centers in China who underwent chest computed tomography (CT) and echocardiography. Skeletal muscle index (SMI) and skeletal muscle density (SMD) were measured by opportunistic chest CT at the first lumbar vertebra level to assess muscle mass and muscle quality. Patients were categorized by calcified valves: no calcification, single-valve (aortic or mitral) calcification, and dual-valve calcification. Ordinal logistic regression assessed the relationships of SMI and SMD with CVC risk. Of 2,140 patients (mean age of 55 years, 58.8% male), 782 (36.5%) exhibited CVC: 550 (25.7%) with single-valve calcification, and 232 (10.8%) with dual-valve calcification. As SMD quartiles decreased, the ORs (95% CIs) for CVC consistently increased (1.22 [0.90–1.64], 1.46 [1.08–1.97], 1.49 [1.07–2.08]; *P* = 0.003) after adjusting for potential confounders. The OR (95% CI) for CVC associated with a 1 SD decrease in SMD was 1.20 (1.06–1.36; *P* = 0.004) in Model 3. Multivariable adjustments revealed no significant links between SMI and CVC risk. In this large multicenter study, we found that low SMD, but not low SMI, is independently associated with CVC in dialysis patients. Integrating SMD assessments into routine care may improve CVC management for this population.

## Introduction

Cardiovascular disease (CVD) is the principal cause of mortality for patients undergoing dialysis (Jankowski et al., 2021; Lai et al., 2021). The prevalence of cardiac valve calcification (CVC) in dialysis patients is eight times higher than that in the general population (Moshar et al., 2016). CVC often causes valvular stenosis and regurgitation, along with complications like conduction system abnormalities and endocarditis, which are major contributors to cardiovascular morbidity and overall mortality (Marwick et al., 2019; Wang et al., 2018). Accurate risk assessment of CVC is crucial for guiding primary prevention strategies.

Sarcopenia, characterized by low muscle mass alongside reduced muscle strength and/or physical performance, has a reported prevalence of 18.1%–55.8% among dialysis patients (Duarte et al., 2024). Sarcopenia is a significant predictor of cardiovascular events (Wathanavasin et al., 2022). Furthermore, several studies have identified associations between sarcopenia and the risks of calcification in different vascular beds, such as coronary artery calcification (CAC), thoracic aortic calcification, and carotid artery intima-media thickness (C-IMT) (Larsen et al., 2024; Lee et al., 2021; Liu et al., 2024; Wang et al., 2025). However, only one study with 106 individuals has mentioned the presence of a relationship between sarcopenia and CVC in elderly subjects (Caffarelli et al., 2023). An increasing body of evidence indicates that processes such as chronic inflammation, insulin resistance, metabolic acidosis, and oxidative stress, which are prevalent in dialysis patients, can contribute to both skeletal muscle wasting and ectopic mineralization within cardiac valves (Sabatino et al., 2021; Urena-Torres et al., 2020). This observation implies a distinct mechanistic link between sarcopenia and CVC. Further investigations are required to clarify the association between muscle abnormalities and valvular calcification in this population.

Computed tomography (CT) is recognized as one of the gold standard options in measuring muscle mass and quality, offering the advantage of providing both quantitative and qualitative data through CT-based analysis (Sabatino et al., 2024). The skeletal muscle index (SMI) from CT scans represents the adjusted area of skeletal muscles, reflecting muscle mass. Skeletal muscle density (SMD) indicates the mean density within this area, reflecting muscle quality (Boutin et al., 2015). Low SMI and SMD have been independently associated with increased postoperative complications, prolonged length of stay, readmissions, and mortality (Xiao et al., 2020; Kays et al., 2020; Giani et al., 2022). Our previous study has confirmed that low SMD measured by CT scan at the first lumbar vertebra (L1) level is an independent predictor of cardiac death in initial-dialysis patients (Sheng et al., 2023), supporting the use of opportunistic chest CT images at L1 for muscle assessment in this population. Therefore, this study aimed to clarify how muscle mass and quality assessed by CT at L1 level relate to the risk of CVC in dialysis patients from four centers in China.

## Methods

### Participants and ethics

**FIGURE 1:**
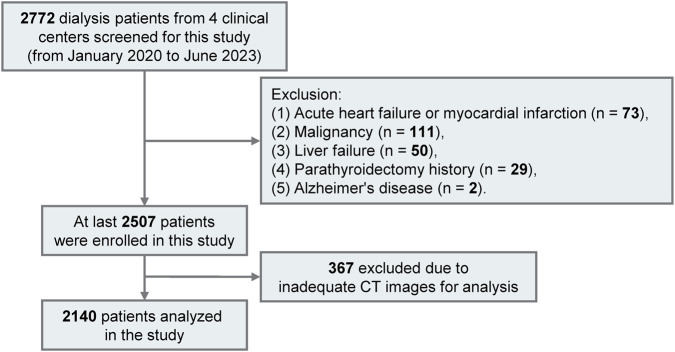
Flowchart of patient selection in the cross-sectional study. From 2,772 dialysis patients, 2,140 were eligible for inclusion.

This study recruited 2,772 patients aged 18 to 80 undergoing regular hemodialysis or peritoneal dialysis at four nephrology and dialysis centers in China from January 2020 to June 2023. All participants received chest non-enhanced multislice CT scans including the L1 level. Patients received echocardiographic evaluations during their hospitalization. All of the above-mentioned imaging and the laboratory measurements described below were completed within 1 month. Excluded patients are detailed in Figure 1. Ethical approval for this research was granted by Zhongda Hospital’s medical research ethics committee (approval number: 2023ZDSYLL172-P01) and it was registered in the Chinese Clinical Trial Registry on 30 August 2023 (registration number: ChiCTR2300075231), adhering to the Declaration of Helsinki. Due to its retrospective design, the study was granted a waiver from requiring signed informed consent.

### Cardiac valve calcification measurements

Two-dimensional echocardiograms were performed on non-dialysis days using the Vivid 7 system (GE Medical Systems, Milwaukee, WI, USA). Adhering to the protocols recommended by the American Society of Echocardiography, the echocardiograms were executed by two independent sonographers who were unaware of additional clinical information (Schiller et al., 1989). The evaluation entailed capturing two-dimensional echocardiographic views of the aortic and mitral valves using parasternal long-axis and short-axis positions, accompanied by continuous wave Doppler ultrasonography. CVC was identified by the observation of bright echoes > 1 mm on any cusps of the aortic valve, mitral valve, or mitral annulus. Based on calcified valve counts, patients were sorted into three groups: (1) no valve calcification; (2) calcification in only one valve, either aortic or mitral; and (3) calcification in both valves (Takahashi et al., 2013). Left ventricular ejection fraction (LVEF) was assessed using the biplane Simpson method by manually tracing the endocardial borders on two-dimensional images.

### CT image analysis for SMI and SMD

Chest CT examinations were performed using the Discovery CT750, Revolution CT, and Optima CT 660 (all from GE Healthcare, Milwaukee, WI), SOMATOM Sensation (Siemens Healthineers, Erlangen, Germany), or Ingenuity CT (Philips, Amsterdam, Netherlands). CT scan parameters in the Supplementary Method 1. We utilized a single axial CT scan at the L1 vertebra to analyze the skeletal muscle using 3D Slicer (version 5.0.3, https://www.slicer.org) (Fedorov et al., 2012). The assessment included skeletal muscle area encompassing the psoas major, erector spinae, quadratus lumborum, transversus abdominis, obliques (both external and internal), and the rectus abdominis. These were evaluated and measured based on attenuation values ranging from −29 to 150 Hounsfield Units (HU). SMI was derived by normalizing the muscle area relative to the patient’s height squared (cm^2^/m^2^), serving as an indicator of skeletal muscle mass. SMD was assessed using the average radiation attenuation value across the entire muscle area at the L1 level.

### Potential covariates

Covariates were identified through literature review and clinical expertise (Zhang et al., 2023; Kaiser et al., 2022). All research data were collected by trained personnel. The data collection processes and equipment were standardized across four research sites. Demographic and clinical data were collected from patient records, including age, sex, height, weight, body mass index (BMI), systolic blood pressure (SBP), diastolic blood pressure (DBP), smoking history, dialysis modality (hemodialysis vs. peritoneal dialysis), dialysis duration, histories of diabetes, hypertension, coronary heart disease (CHD), hyperlipidemia, and stroke, in addition to medication history involving vitamin D, calcium supplements, cinacalcet, and non-calcium-containing phosphate binders. Definitions of diabetes, hypertension, CHD, hyperlipidemia, and stroke are detailed in the Supplementary Method 2. Cigarette smoking was defined as having smoked over 100 cigarettes in one’s lifetime. Since all participants were Chinese, data on race and ethnic categories were not collected.

Levels of white blood cell count (WBC), hemoglobin (Hb), fasting plasma glucose (FPG), triglycerides (TG), total cholesterol (TC), high-density lipoprotein cholesterol (HDL-C), low-density lipoprotein cholesterol (LDL-C), albumin (ALB), aspartate transaminase (AST), alanine transaminase (ALT), gamma-glutamyl transferase (GGT), uric acid (UA), bicarbonate, serum calcium, serum phosphate, and intact parathyroid hormone (iPTH) were measured using standard laboratory methods. The calculation method for corrected serum calcium is described in Supplementary Method 2.

### Statistical analyses

In this analysis, numerical variables were presented as mean (SD) or median (P25, P75). Categorical variables were reported as frequencies (percentages). Given the significant impact of sex on muscle mass and attenuation values, our study performed sex-specific quartile analyses of SMI and SMD for male and female participants (Wang et al., 2024). Characteristics of the study population were compared based on CVC categories and SMD quartiles. Continuous variables were analyzed using one-way ANOVA with Bonferroni method as the post hoc analysis or the Kruskal–Wallis test, and categorical variables were evaluated with the χ^2^ test.

To investigate the association between SMI and SMD (as continuous variables and as categorized variables stratified by sex) with CVC risk among dialysis patients, odds ratios (ORs) and 95% confidence intervals (CIs) were calculated using an ordinal logistic regression model after validating the proportional odds assumption with the Brant test. Three models were gradually adjusted for covariates. Model 1 was adjusted for SMI (SMD model), SMD (SMI model), age, and sex. Model 2 further incorporated BMI, smoking history, dialysis duration, hypertension, and diabetes. Model 3 further incorporated WBC (log WBC), TG [log(TG + 1)], LDL-C, iPTH (log iPTH), serum phosphate, corrected serum calcium, and Vitamin D use. The model 3 was used as our main model. We conducted a receiver operating characteristic (ROC) analysis to evaluate the diagnostic performance of SMD for CVC across the general population and by sex.

We further conducted subgroup analyses stratified by age (<65 or ≥65 years), sex (female or male), BMI (<25 or ≥25 kg/m^2^), smoking (yes or no), diabetes (yes or no), hypertension (yes or no), and dialysis modality (hemodialysis or peritoneal dialysis). *P* for interactions between these covariates and SMD were calculated by entering a multiplication term in model 3. Furthermore, several sensitivity analyses were conducted to test the robustness of our findings. (1) To clarify the intrinsic relationship between SMD and the risk of CVC and reduce potential confounders, patients with a history of stroke and CHD were excluded. (2) To further test the robustness of the association between SMD and CVC, we categorized patients into binary groups based on different SMD cutoff points. (3) To explore the minimum strength of association that any unmeasured confounder would need to fully explain away any association, we calculated the E value using VanderWeele and Ding’s methodology, detailed in Supplementary Method 3; (VanderWeele and Ding, 2017).

All analyses were conducted using R version 4.3.1 (R Foundation for Statistical Computing, Vienna, Austria) or STATA version 16.0 (StataCorp LLC, College Station, TX, USA). Two-sided *P* values <0.05 were considered statistically significant.

## Result

### Characteristics of the subjects according to valve calcification categories

The study involved 2,140 participants with a mean (SD) age of 55 (14) years, including 1,259 males (58.8%) and 881 females (41.2%). The mean BMI (SD) was 23.0 (3.9) kg/m^2^. Among the participants, 1,722 (80.5%) were undergoing hemodialysis and 418 (19.5%) were on peritoneal dialysis, with mean (SD) dialysis duration of 3.5 (4.4) years. A total of 782 patients (36.5%) exhibited CVC: 550 (25.7%) had single-valve calcification (aortic valve only in 463 [21.6%] and mitral valve only in 87 [4.1%]), and 232 (10.8%) had dual-valve calcification. Table 1 outlines the baseline characteristics segmented by valve calcification types. Patients diagnosed with valve calcification tended to be older, exhibited longer dialysis duration, and had higher prevalence of diabetes, CHD, hyperlipidemia, and stroke. They were more likely to use cinacalcet, showed elevated levels of FPG and GGT, and had reduced LVEF.

**TABLE 1:** Characteristics of the Study Population by Number of Calcified Valves (mitral and aortic).

Characteristic	Over all	No. of calcified valves			*P* value
		0	1	2	
Number	2,140	1,358	550	232	
Age, years	55 (14)	51 (14)	61 (12)*	62 (11)*	<0.001
Men, n (%)	1,259 (58.8%)	804 (59.2%)*	332 (60.4%)*	109 (53.0%)*	0.146
Systolic BP, mmHg	145 (26)	145 (15)*	145 (26)*	145 (28)*	0.928
Diastolic BP, mmHg	85 (15)	87 (15)	83 (15)*	81 (16)*	<0.001
BMI, kg/m^2^	23.0 (3.9)	22.9 (3.9)*	23.4 (3.9)*	22.9 (3.8)*	0.085
Smoking history, n (%)	284 (13.3%)	180 (13.3%)*	74 (13.5%)*	30 (12.9%)*	0.980
Dialysis duration, years	3.5 (4.4)	2.9 (3.9)	3.9 (4.6)	6.3 (5.2)	<0.001
Dialysis modality, n (%)					<0.001
Hemodialysis	1722 (80.5%)	1,049 (77.3%)	471 (85.6%)*	202 (87.1%)*	
Peritoneal dialysis	418 (19.5%)	309 (22.8%)	79 (14.4%)*	30 (12.9%)*	
Diabetes, n (%)	676 (31.6%)	351 (25.9%)	230 (41.8%)*	95 (41.0%)*	<0.001
Hypertension, n (%)	1861 (87.0%)	1,159 (85.4%)*	499 (90.7%)†	203 (87.5%)*†	0.007
Coronary heart disease, n (%)	281 (13.1%)	123 (9.1%)	99 (18%)*	59 (25.4%)*	<0.001
Hyperlipidemia, n (%)	397 (18.6%)	205 (15.1%)	135 (24.6%)*	57 (24.6%)*	<0.001
Stroke, n (%)	317 (14.8%)	156 (11.5%)	114 (20.7%)*	47 (20.3%)*	<0.001
Medication history, n (%)					
Vitamin D	1,082 (50.6%)	694 (51.1%)*	267 (48.6%)*	121 (52.2%)*	0.525
Calcium supplements	591 (27.6%)	388 (28.6%)*	142 (25.8%)*	61 (26.3%)*	0.425
Cinacalcet	325 (15.2%)	185 (13.6%)*	89 (16.2%)*†	51 (22%)†	0.003
Non-calcium−containing phosphate binders	949 (44.4%)	594 (43.7%)	249 (45.3%)*	106 (45.7%)*	0.755
Laboratory results					
WBC, *10^9^/L	6.2 (4.9–7.8)	6.1 (4.8–7.6)*	6.3 (5.1–8.0)†	6.2 (5.1–8.0)*†	0.041
Hemoglobin, g/L	98 (22)	97 (23)*	99 (23)*	98 (20)*	0.388
Albumin, g/L	35.0 (5.6)	35.1 (5.7)*	34.9 (5.6)*	34.7 (5.2)*	0.459
FPG, mmol/L	5.2 (4.4–6.9)	5.1 (4.3–6.5)	5.4 (4.5–8.1)*	5.4 (4.5–7.8)*	<0.001
Uric acid, μmol/L	395 (133)	402 (135)	384 (128)*	374 (123)*	0.001
Triglycerides, mmol/L	1.4 (1.0–2.1)	1.4 (1.0–2.1)*	1.4 (1.0–2.1)*	1.3 (1.0–1.8)*	0.164
Total cholesterol, mmol/L	3.8 (1.2)	3.9 (1.2)*	3.8 (1.2)*	3.5 (1.1)	<0.001
HDL cholesterol, mmol/L	1.0 (0.3)	1.0 (0.3)*	1.0 (0.3)*	1.0 (0.3)*	0.215
LDL cholesterol, mmol/L	2.2 (0.9)	2.2 (0.9)	2.1 (0.9)*	1.9 (0.8)*	<0.001
AST, U/L	15 (11–20)	15 (11–20)*	15 (11–19.9)*	16 (11–21)*	0.532
ALT, U/L	11 (7–17)	11 (7–18)*	11 (7.5–16.1)*	11 (7–17)*	0.613
GGT, U/L	21 (14–36)	20 (14–34)	23 (15–36)	27 (17–53)	<0.001
Bicarbonate, mmol/L	22.5 (4.3)	22.6 (4.4)*	22.3 (3.9)*	22.6 (4.2)*	0.413
Corrected serum calcium, mmol/L	2.3 (0.2)	2.3 (0.2)*	2.3 (0.2)*	2.3 (0.3)*	0.285
Serum phosphate, mmol/L	1.8 (0.6)	1.9 (0.6)*	1.8 (0.6)†	1.8 (0.7)*†	0.004
iPTH, pg/mL	243.2 (126.6–440.4)	243.4 (134.5–423.7)*	235.8 (110.0–446.5)*	265.0 (123.9–606.8)*	0.097
LVEF, %	61.9 (10.0)	62.4 (9.4)*	61.2 (10.7)*†	60.6 (11.8)†	0.006
SMD	33.2 (8.4)	34.9 (8.2)	31.2 (8.0)	28.4 (8.2)	<0.001
SMI	38.5 (8.3)	38.8 (8.3)*	38.4 (8.3)*	36.8 (8.0)	0.004

Data are presented as mean (SD), n (%), or median (IQR). BP, indicates blood pressure; BMI, body mass index; WBC, white blood cell count; FPG, fasting plasma glucose; HDL, high-density lipoprotein; LDL, low-density lipoprotein; AST, aspartate aminotransferase; ALT, alanine aminotransferase; GGT, gamma-glutamyl transferase; iPTH, intact parathyroid hormone; LVEF, left ventricular ejection fractions; SMD, skeletal muscle density; SMI, skeletal muscle index.

* † ‡ The same letters indicate a statistically insignificant difference.

Additionally, mean SMD values were 33.2 (SD, 8.4) HU, 31.2 (SD, 8.0) HU, and 28.4 (SD, 8.2) HU for groups with 0, 1, and 2 calcified valves, respectively (*P* < 0.001 for 1 vs. 0 calcified valves; *P* < 0.001 for 2 vs. 0 calcified valves; Table 1). Similarly, mean SMI values were 38.5 (SD, 8.3) cm^2^/m^2^, 38.4 (SD, 8.3) cm^2^/m^2^, and 36.8 (SD, 8.0) cm^2^/m^2^ in these groups (*P* = 1.000 for 1 vs. 0 calcified valves; *P* = 0.003 for 2 vs. 0 calcified valves; Table 1).

### Characteristics of the subjects based on SMD quartiles

**FIGURE 2:**
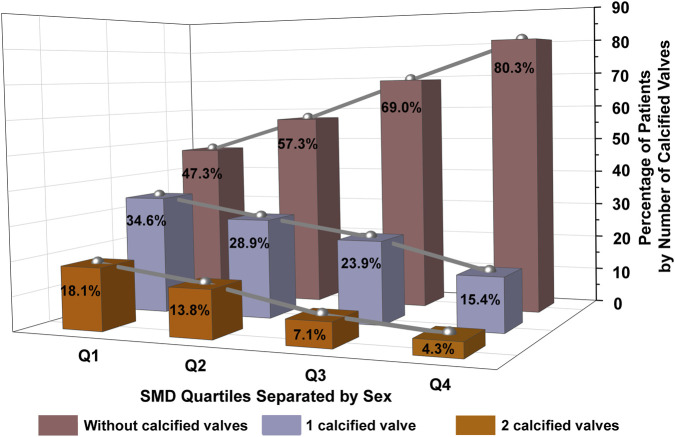
Distribution of calcified valves by SMD quartiles separated by sex. Figure 2 shows the percentage of patients with different numbers of calcified valves across SMD quartiles, separated by sex. Quartiles Q1 to Q4 represent increasing levels of SMD. Different colors of the bars represent the number of calcified valves. The data illustrate that lower SMD quartiles have a higher prevalence of calcified valves, with prevalence decreasing as SMD increases. SMD, skeletal muscle density.

We calculated sex-specific cut points for SMD quartiles and used these to further classify participants (Table 2). Compared to patients in higher SMD quartiles, those in the lowest quartile were typically older, had higher BMI, and higher prevalence of diabetes, hypertension, CHD, hyperlipidemia, and stroke. They also had lower rates of vitamin D use and worse metabolic parameters, including lower levels of Hb, ALB, and HDL, as well as higher levels of WBC, FPG, AST, and GGT. As the quartiles of SMD decreased, the prevalence of both single-valve and dual-valve calcification increased, while the number of individuals without valve calcification decreased (Figure 2).

**TABLE 2:** Baseline characteristics of total subjects according to the SMD quartile separated by sex.

Characteristic	Over all	SMD quartile separated by sex				*P* value
		Q1	Q2	Q3	Q4	
Number	2,140	535	536	535	534	
SMD, HU						
Men	35.1 (8.0)	24.6 (4.3)	32.9 (1.6)	38.0 (1.4)	45.0 (3.9)	
Women	30.5 (8.3)	20.0 (3.9)	27.6 (1.7)	33.5 (1.8)	41.0 (3.9)	
Age, years	55 (14)	66 (11)	58 (11)	52 (12)	44 (12)	<0.001
Systolic BP, mmHg	145 (26)	142 (27)*	146 (26)*	145 (24)*	146 (25)*	0.105
Diastolic BP, mmHg	85 (15)	79 (14)	84 (15)	87 (15)	91 (16)	<0.001
BMI, kg/m^2^	23.0 (3.9)	24.2 (4.0)	23.1 (3.6)*	22.9 (3.8)*	22.0 (3.7)	<0.001
Smoking history, n (%)	284 (13.3%)	75 (14.0%)*	79 (14.7%)*	70 (13.1%)*	60 (11.2%)*	0.362
Dialysis duration, years	3.5 (4.4)	3.4 (4.1)*†	4.0 (5.0)*	3.5 (4.3)*†	3.2 (4.0)†	0.017
Dialysis modality, n (%)						<0.001
Hemodialysis	1722 (80.5%)	485 (90.7%)	450 (84.0%)	408 (76.3%)*	379 (71.0%)*	
Peritoneal dialysis	418 (19.5%)	50 (9.3%)	86 (16.0%)	127 (23.7%)*	155 (29.0%)*	
Diabetes, n (%)	676 (31.6%)	267 (49.9%)	196 (36.6%)	143 (26.7%)	70 (13.1%)	<0.001
Hypertension, n (%)	1861 (87.0%)	481 (89.9%)*	466 (86.9%)*†	471 (88.0%)*†	443 (83.0%)†	0.007
Coronary heart disease, n (%)	281 (13.1%)	131 (24.5%)	80 (14.9%)	46 (8.6%)	24 (4.5%)	<0.001
Hyperlipidemia, n (%)	397 (18.6%)	142 (26.5%)*	111 (20.7%)*†	98 (18.3%)†	46 (8.6%)	<0.001
Stroke, n (%)	317 (14.8%)	141 (26.4%)	88 (16.4%)*	61 (11.4%)*	27 (5.1%)	<0.001
Medication history, n (%)						
Vitamin D	1,082 (50.6%)	248 (46.4%)*	261 (48.7%)*†	278 (52.0%)†	295 (55.2%)†	0.022
Calcium supplements	591 (27.6%)	167 (31.2%)*	167 (31.2%)*	144 (26.9%)*	138 (25.8%)*	0.189
Cinacalcet	325 (15.2%)	72 (13.5%)*	82 (15.3%)*	89 (16.6%)*	82 (15.4%)*	0.546
Non-calcium−containing phosphate binders	949(44.4%)	181 (33.8%)	241 (45.0%)*	248 (46.4%)*	279 (52.3%)*	<0.001
Laboratory results						
WBC, *10^9^/L	6.2 (4.9–7.8)	6.5 (5.1–8.4)†	6.1 (4.9–7.8)*†	6.2 (4.9–7.7)*†	6.0 (4.8–7.3)*	0.003
Hemoglobin, g/L	98 (22)	95 (23)†	97 (22)*†	100 (23)*	99 (22)*	0.001
Albumin, g/L	35.0 (5.6)	34.2 (5.7)*	34.5 (5.6)*†	35.4 (5.8)† ‡	36.1 (5.2)‡	<0.001
FPG, mmol/L	5.2 (4.4–6.9)	5.9 (4.6–8.6)	5.3 (4.4–7.2)	5.0 (4.3–6.7)*	4.9 (4.2–6.0)*	<0.001
Uric acid, μmol/L	395 (133)	385 (135)*	389 (126)*†	397 (129)*†	408 (139)†	0.030
Triglycerides, mmol/L	1.4 (1.0–2.1)	1.4 (1.0–2.1)*	1.4 (1.0–2.1)*	1.4 (1.0–2.0)*	1.4 (1.0–2.0)*	0.966
Total cholesterol, mmol/L	3.8 (1.2)	3.5 (1.2)	3.8 (1.1)	4.0 (1.2)*	4.0 (1.2)*	<0.001
HDL cholesterol, mmol/L	1.0 (0.3)	0.9 (0.3)	1.0 (0.3)*	1.0 (0.3)*†	1.0 (0.3)†	<0.001
LDL cholesterol, mmol/L	2.2 (0.9)	2.0 (0.9)	2.1 (0.8)*	2.3 (0.9)*†	2.3 (0.9)†	<0.001
AST, U/L	15 (11–20)	16 (11–22)†	15 (11–20)*†	15 (11–20)*†	15 (11–19)*	0.033
ALT, U/L	11 (7–17)	11 (7–18)*	11 (7–17)*	11 (7–17)*	11 (7–18)*	0.856
GGT, U/L	21 (14–36)	26 (17–46)*	25 (16–40)*	20 (14–33)	17 (13–28)	<0.001
Bicarbonate, mmol/L	22.5 (4.3)	22.2 (4.2)*	22.2 (4.2)*	22.6 (4.5)*†	23.0 (4.2)†	0.005
Corrected serum calcium, mmol/L	2.3 (0.2)	2.3 (0.2)*	2.3 (0.2)*	2.3 (0.3)*	2.3 (0.2)*	0.384
Serum phosphate, mmol/L	1.8 (0.6)	1.7 (0.6)*	1.8 (0.6)*	1.9 (0.7)†	1.9 (0.6)†	<0.001
iPTH, pg/mL	243.2 (126.6–440.4)	222.3 (106.8–373.2)*	231.1 (117.8–447.4)*†	255.2 (133.8–455.6)†	268.6 (146.8–469.0)*†	0.001
LVEF, %	61.9 (10.0)	61.6 (11.1)*	61.5 (10.3)*	62.2 (10.1)*	62.3 (8.5)*	0.444

Data are presented as mean (SD), n (%), or median (IQR). SMD, indicates skeletal muscle density; BP, blood pressure; BMI, body mass index; WBC, white blood cell count; FPG, fasting plasma glucose; HDL, high-density lipoprotein; LDL, low-density lipoprotein; AST, aspartate aminotransferase; ALT, alanine aminotransferase; GGT, gamma-glutamyl transferase; iPTH, intact parathyroid hormone; LVEF, left ventricular ejection fractions.

* † ‡ The same letters indicate a statistically insignificant difference.

### Association between SMD and valve calcification

**FIGURE 3:**
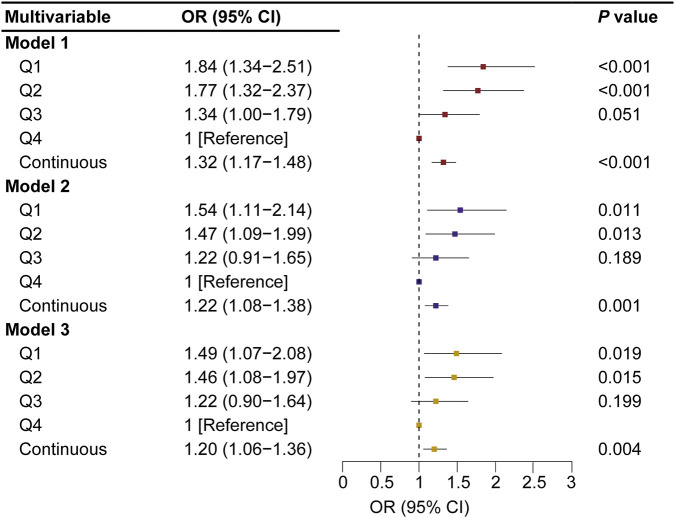
Odds Ratios (95% CIs) of CVC According to SMD Model 1: adjusted for SMI, age, and sex. Model 2: adjusted for all the covariates included in model 1 and additionally adjusted for BMI, smoking history, dialysis duration, hypertension, and diabetes. Model 3: included all the covariates from model 2 and additionally adjusted for WBC (log WBC), TG [log(TG + 1)], LDL-C, iPTH (log iPTH), serum phosphate, corrected serum calcium, and Vitamin D use. CVC indicates cardiac valve calcification; SMD, skeletal muscle density; SMI, skeletal muscle index; BMI, body mass index; WBC, white blood cell count; TG, triglycerides; LDL-C, low-density lipoprotein cholesterol; iPTH, intact parathyroid hormone; CI, confidence interval. The SMD variation is expressed per 1 standard deviation decrease. *P* for trend: Model 1: *P* < 0.001; Model 2: *P* = 0.009; Model 3: *P* = 0.017.

**FIGURE 4:**
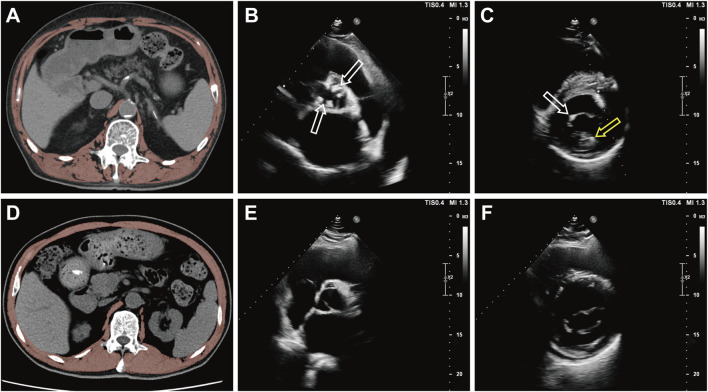
Illustrative Diagram of the Relationship Between SMD and the Presence of Valve Calcification **(A)** A 69-year-old male underwent a chest CT showing L1 SMD = 17.6 HU, **(B)** followed by transthoracic echocardiography in parasternal short-axis view demonstrating aortic valve calcification (white arrow), and **(C)** concurrent calcification of the mitral valve leaflets (white arrow) and mitral valve annulus (yellow arrow). **(D)** Another 69-year-old male with chest CT showing L1 SMD = 49.7 HU, **(E)** transthoracic echocardiography in the same view showing no aortic valve calcification, and **(F)** no calcification in the mitral valve leaflets or annulus. SMD indicates skeletal muscle density; L1, the first lumbar vertebra.

**FIGURE 5:**
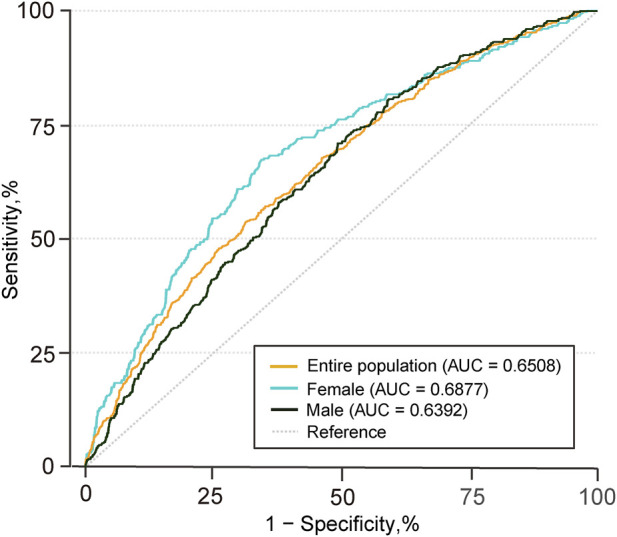
Diagnostic Performance of SMD for CVC Risk in the Entire Population and across Sexes SMD indicates skeletal muscle density; CVC, cardiac valve calcification; AUC, area under the curve. All diagnostic *P*-values are less than 0.001.

To assess the relationship between SMD and the risk of valve calcification, an ordinal logistic regression model was applied, calculating the ORs and 95% CIs for single-valve and dual-valve calcification across SMD (as SMD quartile stratified by sex and as continuous variables). After multivariable adjustment, a significant inverse association was observed between SMD and the risk of valve calcification (Figures 3, 4). In the fully adjusted model (model 3), compared to the reference group (the fourth quartile), the ORs for valve calcification were 1.22 (95% CI, 0.90–1.64; *P* = 0.199) in the third quartile, 1.46 (95% CI, 1.08–1.97; *P* = 0.015; E-value, 2.28 [upper CI, 1.37]) in the second quartile, and 1.49 (95% CI, 1.07–2.08; *P* = 0.019; E-value, 2.34 [upper CI, 1.34]) in the first quartile. When SMD was introduced as a continuous variable in multivariate models, the OR (95% CI) for CVC associated with a 1 SD decrease in SMD was 1.20 (1.06–1.36; *P* = 0.004; Figure 3, model 3). Restricted cubic spline analysis indicated that lower SMD levels were associated with a higher risk of CVC, and no significant evidence of nonlinearity was detected (*P* for nonlinearity = 0.579) (Supplementary Figure S1). After multivariable adjustment, SMI quartiles and continuous SMI values showed no significant association with the risk of valve calcification (Supplementary Table S1). ROC analysis demonstrated that SMD has predictive capability for valve calcification among the general population and sex-specific subgroups, with AUC values of 0.651 for the overall group (*P* < 0.001), 0.639 for males (*P* < 0.001), and 0.688 for females (*P* < 0.001) as shown in Figure 5. The overall optimal cut-off value for SMD was 31.16 HU, corresponding to the maximum Youden index (0.223), with a sensitivity of 53.7% and specificity of 68.6%.

### Subgroup and sensitivity analyses

As shown in Table 3, subgroup analyses revealed that most ORs of valve calcification risk were statistically significant within subgroups. After adjusting for multiple testing, no significant interactions were observed between SMD or any stratified variables with the risk of valve calcification (all *P* for interaction >0.05).

**TABLE 3:** Odds ratios (95% CIs) for the association between SMD and CVC in clinically relevant subgroups.

Subgroup	SMD quartile separated by sex				*P*−t	Continuous variable (per 1 SD decrease in SMD)	*P* value	*P*−int
	Q1 (lowest)	Q2	Q3	Q4 (highest)				
Age								0.340
<65	1.61 (1.07–2.42)	1.37 (0.97–1.93)	1.22 (0.88–1.69)	1 (ref.)	0.017	1.23 (1.07–1.41)	0.005	
≥65	1.34 (0.61–2.93)	1.48 (0.67–3.28)	1.09 (0.47–2.51)	1 (ref.)	0.429	1.12 (0.93–1.34)	0.242	
Sex								0.059
Female	1.72 (1.01–2.94)	1.61 (1.00–2.58)	0.96 (0.60–1.54)	1 (ref.)	0.015	1.34 (1.10–1.63)	0.004	
Male	1.37 (0.89–2.11)	1.41 (0.94–2.10)	1.40 (0.94–2.07)	1 (ref.)	0.288	1.13 (0.97–1.32)	0.109	
BMI								0.762
<25	1.55 (1.05–2.31)	1.58 (1.12–2.24)	1.18 (0.84–1.66)	1 (ref.)	0.015	1.22 (1.06–1.41)	0.007	
≥25	1.30 (0.67–2.52)	1.16 (0.61–2.22)	1.44 (0.75–2.76)	1 (ref.)	0.705	1.14 (0.90–1.45)	0.290	
Smoking								0.952
Yes	1.50 (0.59–3.78)	1.37 (0.58–3.23)	1.40 (0.58–3.38)	1 (ref.)	0.474	1.21 (0.87–1.69)	0.265	
No	1.49 (1.04–2.14)	1.48 (1.07–2.05)	1.21 (0.88–1.67)	1 (ref.)	0.024	1.20 (1.05–1.38)	0.008	
Diabetes								0.354
Yes	1.03 (0.56–1.89)	1.05 (0.58–1.89)	0.94 (0.51–1.75)	1 (ref.)	0.797	1.07 (0.88–1.30)	0.507	
No	1.82 (1.20–2.76)	1.65 (1.15–2.37)	1.34 (0.95–1.90)	1 (ref.)	0.004	1.30 (1.11–1.52)	0.001	
Hypertension								0.261
Yes	1.44 (1.01–2.06)	1.36 (0.98–1.88)	1.26 (0.92–1.74)	1 (ref.)	0.060	1.17 (1.02–1.33)	0.023	
No	2.08 (0.76–5.64)	2.42 (1.04–5.64)	0.87 (0.34–2.26)	1 (ref.)	0.057	1.59 (1.07–2.35)	0.021	
Dialysis modality								0.742
Hemodialysis	1.50 (1.04–2.17)	1.48 (1.06–2.08)	1.18 (0.84–1.66)	1 (ref.)	0.022	1.22 (1.40–1.07)	0.003	
Peritoneal dialysis	1.32 (0.57–3.07)	1.34 (0.65–2.76)	1.38 (0.73–2.62)	1 (ref.)	0.547	1.07 (1.47–0.78)	0.661	

The model used in the subgroups analysis consisted of all covariates used in Model 3 except for the variables that were used for stratification. The odds ratio was examined regarding Q4 as reference. *P* for interactions between these covariates and SMD, were calculated by entering a multiplication term in model 3. CI, indicates confidence interval; SMD, skeletal muscle density; CVC, cardiac valve calcification; *P*-t, *P* for trend; *P*-int, *P* for interaction; SD, standard deviation; BMI, body mass index.

Sensitivity analysis results are presented in Supplementary Tables S2–S4. First, after excluding patients with stroke and CHD, SMD remained independently associated with valve calcification in the fully adjusted ordinal logistic regression models (Supplementary Tables S2, S3). Second, SMD was categorized into several binary groups based on optimal cutoff values determined from time-dependent ROC curves (Sheng et al., 2023), median, and mean values. Compared with participants in the higher SMD group, there was a significantly higher risk of valve calcification for participants in the lower group (Supplementary Table S4). Last, to assess the impact of unobservable variables on the effect estimates, E-values were calculated. All E-values were greater than 1, which suggested that considerable unmeasured confounding would be needed to explain away the effect estimate. Details on E-values are provided in the Supplementary Method 3.

## Discussion

CVC is widely observed in dialysis patients and is closely associated with CVD, highlighting the importance of exploring its potential related factors. To our knowledge, this study represents the first multicenter retrospective analysis exploring the associations of skeletal muscle mass and skeletal muscle quality with CVC in dialysis patients. Low SMD at the L1 level, as assessed by CT, was significantly associated with both the presence and severity of CVC. This relationship is independent of conventional cardiovascular risk factors and consistently observed across various subgroups.

CVC primarily affects the mitral and aortic valves. Simple calcifications, though clinically silent, often lead to limited leaflet mobility, obstructed aortic valve openings, and reduced valvular orifice area. CVC is an independent risk factor for myocardial ischemia (Choi et al., 2013). Moreover, CVC is associated with severe cardiovascular events such as arrhythmias, congestive heart failure, and even sudden cardiac death (Marwick et al., 2019). Recent meta-analyses indicate that the presence of CVC increases the risk of all-cause mortality in dialysis patients by 1.6 times and the risk of cardiovascular mortality by 2.4 times (Wang et al., 2018; Chiu et al., 2024). The progressive decline in renal function triggers a cascade of consequences, including elevated parathyroid hormone levels, increased calcium-phosphate products, and excessive levels of 1,25-dihydroxyvitamin D, which significantly exacerbate the onset and progression of CVC (Abd Alamir et al., 2015). Consequently, chronic kidney disease patients experience CVC approximately 10–20 years earlier than the general population, and those with end-stage renal disease, especially those undergoing dialysis, may have a CVC progression rate up to 10 times faster than those in the general population (Marwick et al., 2019; Urena-Torres et al., 2020). A meta-analysis involving 3,376 dialysis patients reported a CVC prevalence ranging from 23.5% to 57.6% (Chiu et al., 2024). According to a nationwide multicenter prospective cohort study in China, the prevalence of CVC among dialysis patients is 29.0% (Zhang et al., 2023). In our study, the prevalence of CVC in dialysis patients was 36.5%, which is consistent with previous findings. In our earlier research, we discovered that initial-dialysis patients with low L1 SMD had a higher risk of cardiovascular mortality (Sheng et al., 2023). Given the high prevalence of CVC in dialysis patients and its detrimental impact on cardiovascular outcomes, we further analyzed the relationship between SMD and CVC in dialysis patients.

Muscle quality encompasses micro- and macroscopic alterations in muscle structure and composition, as well as the functional capacity per unit of muscle mass (Cruz-Jentoft et al., 2019). During muscle atrophy, there is a reduction in muscle mass accompanied by increasing intramuscular fat and fibrous tissue, indicating a decline in muscle quality (Akamatsu et al., 2022). In a study involving 100 diabetic individuals, low-density muscle area in the mid-thigh and muscle attenuation (indicative of high intramuscular fat content) were found to independently correlate with increased C-IMT (Kim et al., 2010). Jensky et al. analyzed the relationship between abdominal lean muscle and vascular calcification across multiple vascular beds, finding that a higher proportion of abdominal lean muscle was associated with lower thoracic aortic calcification, independent of visceral adipose tissue (Jensky et al., 2011). Additionally, multiple studies have confirmed an inverse relationship between muscle quality and the risk of CAC across various populations (Larsen et al., 2024; Lee et al., 2021; Terry et al., 2017). However, there is currently no data on the association between muscle quality and CVC in dialysis patients. Our study provides evidence for the first time of an independent negative correlation between SMD and CVC in dialysis patients. Our finding provides new insights into the factors associated with CVC in dialysis patients and establishes a foundation for further exploration of its underlying mechanisms. Since muscle quality is reversible, improving muscle quality may help reduce the risk of CVC and potentially improve cardiovascular outcomes in dialysis patients. In addition to its association with CVC, SMD also showed statistically significant discriminatory ability in ROC analysis, with an optimal cut-off of 31.16 HU. Importantly, SMD can be obtained directly from routine chest CT examinations that are already widely performed in dialysis care. This makes SMD a practical imaging marker that could help clinicians recognize patients who may be at higher risk for CVC. Incorporating SMD evaluation into routine workflow has the potential to support earlier risk stratification and inform more individualized management strategies in this population.

Poor muscle quality, characterized by muscle fat infiltration, may influence CVC through several mechanisms. First, skeletal muscle is the primary organ for insulin uptake. Intermuscular lipid accumulation in maintenance hemodialysis patients is positively correlated with insulin resistance (Wang et al., 2013). Insulin resistance leads to chronic hyperglycemia and hyperlipemia, resulting in increased risk of CVC (Selig et al., 2022). Furthermore, skeletal muscle fat infiltration is linked to an increase in oxidative stress. Oxidative stress plays a role in the early stages of CVC, driving the transformation of valvular interstitial cells into osteoblast-like cells (Miller et al., 2008). Lastly, muscle fat infiltration is associated with a heightened systemic inflammatory response, which is involved in the CVC process (The et al., 2022).

It has been reported that a negative correlation between appendicular skeletal muscle mass and CVC, suggesting protective effects of higher muscle mass against CVC (Caffarelli et al., 2023). Despite the known associations between low muscle mass and CVD (Jun et al., 2021; Ko et al., 2016), there was no significant association between SMI and CVC in this study. The following factors could explain this discrepancy. Firstly, SMI in some studies is obtained by dual-energy X-ray absorptiometry or bioelectrical impedance analysis, methods that are less accurate than CT in assessing muscle mass (Sabatino et al., 2024). Secondly, increasing evidence suggests that SMI may not accurately reflect cardiovascular risk. A Multi-Ethnic Study of Atherosclerosis (MESA) found that a larger abdominal muscle area was associated with a higher risk CAC profile (larger CAC volume and lower CAC density) (Crawford et al., 2020). Another study found a significant positive correlation between male muscle area and coronary artery disease (Larsen et al., 2024). This might be explained by larger muscle size could be the result of increased fat infiltration. In addition, multiple studies in dialysis patients show no association between SMI and poor outcomes, possibly because the characteristics of muscle mass changes in dialysis differ from those associated with aging or cancer (Sheng et al., 2023; Isoyama et al., 2014; Kittiskulnam et al., 2017).

Our study has several limitations. First, as a cross-sectional study, it limits causal inferences regarding the relationship between muscle quality and the risk of CVC, as well as the exploration of potential pathophysiological mechanisms. Future longitudinal studies with repeated CT scans or interventional trials targeting improvements in SMD (e.g., exercise or nutritional programs) are needed to clarify causality. Second, we did not assess skeletal muscle function (e.g., grip strength, walking speed), with muscle density being a key factor influencing muscle strength. Third, we did not investigate all confounding factors that may impact SMD or CVC, such as education level, income, exercise intensity and duration, and dietary health. To address this, we calculated E-values to evaluate the influence of unmeasured variables on estimated effects to minimize bias. Fourth, although key acquisition parameters were standardized across centers to minimize inter-scanner heterogeneity, the possibility of residual variability cannot be entirely excluded. Finally, as our research was conducted among dialysis patients in four comprehensive hospitals in Eastern China, the results may not extend to different demographic or ethnic populations.

## Conclusion

In summary, our research demonstrated that low SMD, which reflects muscle quality assessed with opportunistic CT scans, is independently associated with the presence and severity of CVC in dialysis patients. These findings suggest that poor muscle quality may indicate a higher burden of valvular calcification in this population. Opportunistic assessment of muscle quality on routine CT scans may therefore provide additional value for cardiovascular evaluation among dialysis patients.

## Data Availability

The raw data supporting the conclusions of this article will be made available by the authors, without undue reservation.

## Glossary

CVD: cardiovascular disease
CVC: cardiac valve calcification
CAC: coronary artery calcification
C-IMT: carotid artery intima-media thickness
CT: computed tomography
SMI: skeletal muscle index
SMD: skeletal muscle radiodensity
L1: the first lumbar vertebra
SBP: systolic blood pressure
DBP: diastolic blood pressure
CHD: coronary heart disease
BMI: body mass index
WBC: white blood cell count
Hb: hemoglobin
FPG: fasting plasma glucose
TG: triglycerides
TC: total cholesterol
HDL-C: high-density lipoprotein cholesterol
LDL-C: low-density lipoprotein cholesterol
ALB: albumin
AST: aspartate transaminase
ALT: alanine transaminase
GGT: gamma-glutamyl transferase
UA: uric acid
iPTH: intact parathyroid hormone
ORs: odds ratios
CIs: confidence intervals
