# Supplementary material for: Low skeletal muscle density is independently associated with cardiac valve calcification in dialysis patients
Source: Front Physiol. 2025 Dec 18;16:1690904. doi: 10.3389/fphys.2025.1690904 (PMC12756121; doi:10.3389/fphys.2025.1690904)
Supplement: Supplementary file 1 [file Table1.docx]

**Supplementary materials**

**Method S1.** CT Scan Parameters

**Method S2.** Covariates Collection

**Method S3.** Sensitivity Analysis for Unmeasured Confounding

**Table S1**. Odds Ratios (95% CIs) of CVC According to SMI

**Table S2.** Odds Ratios (95% CIs) of CVC According to SMD After Excluding Patients Diagnosed with Stroke

**Table S3.** Odds Ratios (95% CIs) of CVC According to SMD After Excluding Patients Diagnosed with Stroke or Coronary Heart Disease

**Table S4.** Multivariable Analysis of the Association Between Groups Defined by Different SMD Cut-points and CVC

**Figure S1.** Restricted Cubic Spline Curve for SMD in Relation to CVC Risk

**Supplementary materials**

**Method S1.** CT Scan Parameters

All CT examinations were performed with the following parameters: 120 kVp; automated dose modulation using automA and smartmA for GE Healthcare machines, CareDose 4D for Siemens Healthineers, and DoseRight for Philips; matrix 512×512; collimation of 0.625 mm; slice thickness of 5 mm.

**Method S2.** Potential Covariates

Diabetes was identified by a fasting plasma glucose level at or above 126mg/dL (to convert glucose to mmol/L, multiply by 0.0555), a nonfasting plasma glucose level at or above 200 mg/dL, or through self-reported diabetes medication use. Blood pressure was measured in the supine position after a rest of 15-20 minutes, taken from the arm without arteriovenous fistula or shunt. Hypertension was defined as a systolic pressure greater than 140 mm Hg, a diastolic pressure greater than 90 mm Hg, or current use of antihypertensive medications. Coronary heart disease was defined as any condition including angina, myocardial infarction, coronary artery stenosis, percutaneous coronary intervention, or coronary artery bypass surgery. Dyslipidemia was characterized by having total cholesterol levels of 5.2 mmol/L (200 mg/dL) or higher, and LDL-cholesterol levels of 3.5 mmol/L (135 mg/dL) or higher, or by being uncontrolled despite treatment with the highest tolerated doses of other lipid-lowering medications for at least four weeks. Stroke was identified by the sudden appearance of a neurological deficit, headache, or other nonvascular symptoms, confirmed by a clinically significant lesion on brain imaging lasting more than 24 hours, or by death occurring within 24 hours.

Corrected serum calcium = total serum calcium (mmol/L) + 0.02 [40 (g/L) – albumin (g/L)] (British medical journal, 1977;1(6061):598).

**Method S3.** Sensitivity Analysis for Unmeasured Confounding

Additional sensitivity analysis was conducted using the E-value methodology of VanderWeele and Ding (Annals of Internal Medicine, 2017 Aug 15;167(4):268−74), which is a measure of whether the inclusion of further confounders is likely to lead to the attenuation of results. This method calculates the minimum strength of association that an unmeasured confounder would need with SMD and the risk of CVC to overcome the statistically significant effect observed in our study where residual confounding is considered a potential problem. This calculation is derived from the OR obtained from our fully adjusted models.

For the current study, low SMD was significantly associated with an increased risk of CVC, as reflected by OR values above 1. Specifically, the OR of SMD in the second quartile was 1.46 (95% CI, 1.08–1.97; *P* = 0.015) and in the first quartile was 1.49 (95% CI, 1.07–2.08; *P* = 0.019) **(Figure 3 of the main article)**. The E-values for the point estimates were 2.28 for the second quartile and 2.34 for the first quartile, with upper confidence interval limits of 1.37 for the second quartile, and 1.34 for the first quartile. Therefore, taking the second quartile as an example, following the methodology outlined by VanderWeele and Ding, the observed OR of 1.46 could be explained by an unmeasured confounder that was associated with both decreases in SMD and increased risk of CVC by a risk ratio of approximately 2.28 each. Moreover, above-mentioned E-values suggest that only stronger unmeasured confounding could explain these associations, yet it is unlikely that such substantial confounding exists in our study.

**Table S1.** Odds Ratios (95% CIs) of CVC According to SMI

|  | **Model 1** | |  | **Model 2** | |  | **Models 3** | |
| --- | --- | --- | --- | --- | --- | --- | --- | --- |
|  | **OR (95% CI)** | ***P* value** |  | **OR (95% CI)** | ***P* value** |  | **OR (95% CI)** | ***P* value** |
| Q1 | 1.12 (0.87−1.46) | 0.374 |  | 1.25 (0.92−1.70) | 0.155 |  | 1.22 (0.89−1.66) | 0.210 |
| Q2 | 1.10 (0.85−1.42) | 0.474 |  | 1.21 (0.91−1.60) | 0.196 |  | 1.20 (0.90−1.59) | 0.214 |
| Q3 | 1.09 (0.84−1.41) | 0.530 |  | 1.10 (0.84−1.44) | 0.493 |  | 1.11 (0.85−1.46) | 0.449 |
| Q4 | 1 (ref.) | − |  | 1 (ref.) | − |  | 1 (ref.) | − |
| *P* for trend | − | 0.481 |  | − | 0.254 |  | − | 0.240 |
| Continuous variable  (Per 1 SD decrease in SMI) | 1.03 (0.93−1.15) | 0.521 |  | 1.07 (0.95−1.22) | 0.263 |  | 1.06 (0.93−1.20) | 0.389 |

Model 1: adjusted for SMD, age, and sex. Model 2: adjusted for all the covariates included in model 1 and additionally adjusted for BMI, smoking history, dialysis duration, hypertension, and diabetes. Model 3: included all the covariates from model 2 and additionally adjusted for WBC (log WBC), TG [log (TG + 1)], LDL-C, iPTH (log iPTH), serum phosphate, corrected serum calcium, and Vitamin D use.

CVC indicates cardiac valve calcification; SMD, skeletal muscle density; SMI, skeletal muscle index; BMI, body mass index; WBC, white blood cell count; TG, triglycerides; LDL-C, low-density lipoprotein cholesterol; iPTH, intact parathyroid hormone; CI, confidence interval; SD, standard deviation.

The SMI variation is expressed per 1 standard deviation decrease.

**Table S2.** Odds Ratios (95% CIs) of CVC According to SMD After Excluding Patients Diagnosed with Stroke

|  | **Model 1** | |  | **Model 2** | |  | **Models 3** | |
| --- | --- | --- | --- | --- | --- | --- | --- | --- |
|  | **OR (95% CI)** | ***P* value** |  | **OR (95% CI)** | ***P* value** |  | **OR (95% CI)** | ***P* value** |
| Q1 | 1.68 (1.20−2.36) | 0.003 |  | 1.44 (1.00−2.06) | 0.048 |  | 1.40 (0.97−2.01) | 0.072 |
| Q2 | 1.63 (1.20−2.23) | 0.002 |  | 1.39 (1.00−1.91) | 0.047 |  | 1.39 (1.00−1.92) | 0.048 |
| Q3 | 1.32 (0.97−1.79) | 0.077 |  | 1.21 (0.89−1.66) | 0.231 |  | 1.21 (0.88−1.66) | 0.243 |
| Q4 | 1 (ref.) | − |  | 1 (ref.) | − |  | 1 (ref.) | − |
| *P* for trend | − | 0.002 |  | − | 0.044 |  | − | 0.066 |
| Continuous variable  (Per 1 SD Decrease in SMD) | 1.27 (1.12−1.44) | < 0.001 |  | 1.19 (1.04−1.36) | 0.012 |  | 1.17 (1.02−1.34) | 0.022 |

Model 1: adjusted for SMI, age, and sex. Model 2: adjusted for all the covariates included in model 1 and additionally adjusted for BMI, smoking history, dialysis duration, hypertension, and diabetes. Model 3: included all the covariates from model 2 and additionally adjusted for WBC (log WBC), TG [log (TG + 1)], LDL-C, iPTH (log iPTH), serum phosphate, corrected serum calcium, and Vitamin D use.

CVC indicates cardiac valve calcification; SMD, skeletal muscle density; SMI, skeletal muscle index; BMI, body mass index; WBC, white blood cell count; TG, triglycerides; LDL-C, low-density lipoprotein cholesterol; iPTH, intact parathyroid hormone; CI, confidence interval; SD, standard deviation.

The SMD variation is expressed per 1 standard deviation decrease.

**Table S3.** Odds Ratios (95% CIs) of CVC According to SMD After Excluding Patients Diagnosed with Stroke or Coronary Heart Disease

|  | **Model 1** | |  | **Model 2** | |  | **Models 3** | |
| --- | --- | --- | --- | --- | --- | --- | --- | --- |
|  | **OR (95% CI)** | ***P* value** |  | **OR (95% CI)** | ***P* value** |  | **OR (95% CI)** | ***P* value** |
| Q1 | 1.79 (1.24−2.59) | 0.002 |  | 1.54 (1.04−2.28) | 0.031 |  | 1.52 (1.02−2.25) | 0.037 |
| Q2 | 1.70 (1.23−2.36) | 0.001 |  | 1.45 (1.03−2.04) | 0.033 |  | 1.46 (1.04−2.06) | 0.030 |
| Q3 | 1.40 (1.02−1.93) | 0.039 |  | 1.28 (0.92−1.79) | 0.142 |  | 1.28 (0.92−1.79) | 0.147 |
| Q4 | 1 (ref.) | − |  | 1 (ref.) | − |  | 1 (ref.) | − |
| *P* for trend | − | 0.001 |  | − | 0.029 |  | − | 0.034 |
| Continuous variable  (Per 1 SD Decrease in SMD) | 1.31(1.15−1.50) | < 0.001 |  | 1.22 (1.06−1.42) | 0.006 |  | 1.22 (1.05−1.41) | 0.008 |

Model 1: adjusted for SMI, age, and sex. Model 2: adjusted for all the covariates included in model 1 and additionally adjusted for BMI, smoking history, dialysis duration, hypertension, and diabetes. Model 3: included all the covariates from model 2 and additionally adjusted for WBC (log WBC), TG [log (TG + 1)], LDL-C, iPTH (log iPTH), serum phosphate, corrected serum calcium, and Vitamin D use.

CVC indicates cardiac valve calcification; SMD, skeletal muscle density; SMI, skeletal muscle index; BMI, body mass index; WBC, white blood cell count; TG, triglycerides; LDL-C, low-density lipoprotein cholesterol; iPTH, intact parathyroid hormone; CI, confidence interval; SD, standard deviation.

The SMD variation is expressed per 1 standard deviation decrease.

**Table S4.** Multivariable Analysis of the Association Between Groups Defined by Different SMD Cut-points and CVC

|  | **Model 1** | |  | **Model 2** | |  | **Models 3** | |
| --- | --- | --- | --- | --- | --- | --- | --- | --- |
|  | **OR (95% CI)** | ***P* value** |  | **OR (95% CI)** | ***P* value** |  | **OR (95% CI)** | ***P* value** |
| Normal SMD vs. Low SMD **^a^** | 1.22 (1.11−1.35) | < 0.001 |  | 1.15 (1.04−1.28) | 0.007 |  | 1.14 (1.03−1.27) | 0.013 |
| Normal SMD vs. Low SMD **^b^** | 1.50 (1.22−1.84) | < 0.001 |  | 1.32 (1.06−1.63) | 0.012 |  | 1.30 (1.04−1.61) | 0.019 |
| Normal SMD vs. Low SMD **^c^** | 1.52 (1.24−1.86) | < 0.001 |  | 1.33 (1.07−1.64) | 0.009 |  | 1.30 (1.05−1.62) | 0.016 |

Model 1: adjusted for SMI, age, and sex. Model 2: adjusted for all the covariates included in model 1 and additionally adjusted for BMI, smoking history, dialysis duration, hypertension, and diabetes. Model 3: included all the covariates from model 2 and additionally adjusted for WBC (log WBC), TG [log (TG + 1)], LDL-C, iPTH (log iPTH), serum phosphate, corrected serum calcium, and Vitamin D use.

^a^ Low SMD, defined as SMD < 39.56 HU for males and < 33.06 HU for females (determined by time-dependent ROC curves based on all-cause mortality from our previous research), was used as the reference to examine the OR;

^b^ Low SMD, defined as SMD < 35.70 HU for males and < 30.45 HU for females (median), was used as the reference to examine the OR;

^c^ Low SMD, defined as SMD < 35.16 HU for males and < 30.52 HU for females (mean), was used as the reference to examine the OR.

CVC indicates cardiac valve calcification; SMD, skeletal muscle density; SMI, skeletal muscle index; BMI, body mass index; WBC, white blood cell count; HDL, high-density lipoprotein; FPG, fasting plasma glucose; iPTH, intact parathyroid hormone; CI, confidence interval; OR, odds ratio.


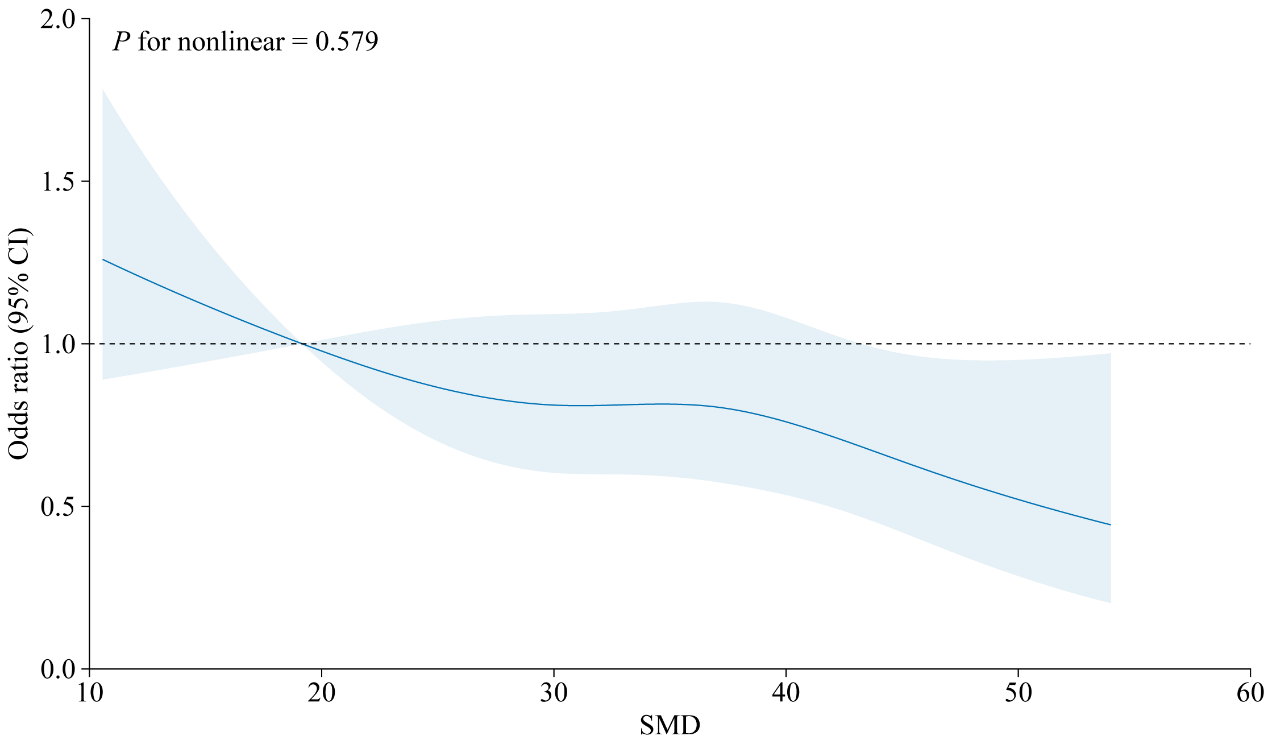


**Figure S1. Restricted Cubic Spline Curve for SMD in Relation to CVC Risk**

The figure illustrates the fully adjusted odds ratios for the association between SMD and CVC risk. Estimates were derived from models adjusted for SMI, age, sex, BMI, smoking history, dialysis duration, hypertension, diabetes, WBC (log WBC), TG [log (TG + 1)], LDL-C, iPTH (log iPTH), serum phosphate, corrected serum calcium, and Vitamin D use. The shaded area represents the 95% confidence interval. CVC indicates cardiac valve calcification; SMD, skeletal muscle density; SMI, skeletal muscle index; BMI, body mass index; WBC, white blood cell count; TG, triglycerides; LDL-C, low-density lipoprotein cholesterol; iPTH, intact parathyroid hormone.
